# Patient, physician, and policy factors underlying variation in use of telemedicine for radiation oncology cancer care

**DOI:** 10.1002/cam4.4555

**Published:** 2022-03-16

**Authors:** Brian De, Shuangshuang Fu, Ying‐Shiuan Chen, Prajnan Das, Kimberly Ku, Sean Maroongroge, Kristina D. Woodhouse, Karen E. Hoffman, Quynh‐Nhu Nguyen, Valerie K. Reed, Aileen B. Chen, Albert C. Koong, Benjamin D. Smith, Grace L. Smith

**Affiliations:** ^1^ Department of Radiation Oncology The University of Texas MD Anderson Cancer Center Houston Texas USA; ^2^ Department of Health Services Research The University of Texas MD Anderson Cancer Center Houston Texas USA

**Keywords:** COVID‐19 pandemic, disparities, policy, radiotherapy, telemedicine

## Abstract

**Background:**

Oncology telemedicine was implemented rapidly after COVID‐19. We examined multilevel correlates and outcomes of telemedicine use for patients undergoing radiotherapy (RT) for cancer.

**Methods:**

Upon implementation of a telemedicine platform at a comprehensive cancer center, we analyzed 468 consecutive patient RT courses from March 16, 2020 to June 1, 2020. Patients were categorized as using telemedicine during ≥1 weekly oncologist visits versus in‐person oncologist management only. Temporal trends were evaluated with Cochran‐Armitage tests; chi‐squared test and multilevel multivariable logistic models identified correlates of use and outcomes.

**Results:**

Overall, 33% used telemedicine versus 67% in‐person only oncologist management. Temporal trends (*p*_trend_ < 0.001) correlated with policy changes: uptake was rapid after local social‐distancing restrictions, reaching peak use (35% of visits) within 4 weeks of implementation. Use declined to 15% after national “Opening Up America Again” guidelines. In the multilevel model, patients more likely to use telemedicine were White non‐Hispanic versus Black or Hispanic (odds ratio [OR] = 2.20, 95% confidence interval [CI] 1.03–4.72; *p* = 0.04) or receiving ≥6 fractions of RT versus 1–5 fractions (OR = 4.49, 95% CI 2.29–8.80; *p* < 0.001). Model intraclass correlation coefficient demonstrated 43% utilization variation was physician‐level driven. Treatment toxicities and 30‐day emergency visits or unplanned hospitalizations did not differ for patients using versus not using telemedicine (*p* > 0.05, all comparisons).

**Conclusion:**

Though toxicities were similar with telemedicine oncology management, there remained lower uptake among non‐White patients. Continuing strategies for oncology telemedicine implementation should address multilevel patient, physician, and policy factors to optimize telemedicine's potential to surmount—and not exacerbate—barriers to quality cancer care.

## Graphical abstract

In this analysis of 468 consecutive patients undergoing radiotherapy after the COVID‐19 pandemic‐driven implementation of an audiovisual telemedicine platform at a large comprehensive cancer center, non‐White patients were less likely to use telemedicine during acute cancer treatment, and 43% of the variation in use patterns was physician‐driven. The risk of treatment toxicities and 30‐day emergency visits or unplanned hospitalizations did not differ by use vs. no use of telemedicine.

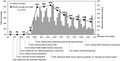

## 1 INTRODUCTION

The COVID‐19 pandemic profoundly altered patterns of in‐person cancer care delivery. Given the vulnerability of cancer patients to severe acute respiratory syndrome coronavirus 2 (SARS‐CoV‐2) and higher risks of serious illness, hospitalization, and death,^1^ use of telemedicine platforms to deliver oncology care increased rapidly to provide continuity of care while minimizing the risk of cancer patients' exposure to COVID‐19.^2^ The movement toward virtual care was particularly marked in the radiation oncology care delivery setting, since radiation oncology care is characterized by a high density of in‐person interactions during acute treatment. A typical radiotherapy (RT) course consists of daily treatments lasting ≤1–7 weeks; until the COVID‐19 pandemic, a treatment course was also standardly accompanied by weekly in‐person visits with radiation oncologists to manage treatment toxicities. As a result of the pandemic, in‐person visits for many oncology practices began to shift to virtual care.

Accordingly, analysis of radiation oncology care delivery in the peri‐pandemic era now represents a distinctly rich, high‐density data source on oncology telemedicine patient‐provider visits that is useful for examining variations in telemedicine uptake and for informing ongoing efforts to refine practice, especially given that oncology telemedicine is forecasted for wide use, even beyond the pandemic.^3^, ^4^, ^5^, ^6^ These data are also critical for early assessment of whether remote management during treatment increases the risk of acute treatment‐related toxicity.

Although several early pandemic studies surveying oncology physicians and patients suggested that telemedicine was associated with high levels of user satisfaction, increased efficiency, and improved continuity of care,^7^, ^8^, ^9^, ^10^, ^11^, ^12^ other studies also identified potential drawbacks—limited physical examinations, patient perceptions of lower quality of care, and lack of patient access to or comfort with the technologies.^13^, ^14^, ^15^, ^16^ Thus, it remains to be seen whether future iterations of cancer telemedicine technologies and practice will effectively and strategically leverage its potential strengths to improve access to and equity in cancer care delivery—or whether its limitations may perpetuate disparities in care delivery.

An evidence gap remains regarding the patterns and predictors of oncology telemedicine uptake and its effects on clinical outcomes.^17^ Data from prior studies of use of molecular testing and enrollment on clinical trials have demonstrated significant variation in their uptake, driven by both patient and physician characteristics.^18^, ^19^, ^20^ Variation and disparities in cancer care delivery have been correlated with both patient and physician characteristics,^18^, ^19^, ^20^ as well as poorer outcomes for vulnerable sociodemographic groups (minority race/ethnicity, female, and the elderly) such as guideline‐discordant care and higher mortality.^21^, ^22^ Therefore, in the face of telemedicine expansion in cancer care, evaluation of the initial variation in and outcomes of oncology telemedicine use is necessary to facilitate understanding of telemedicine's potential limitations and barriers to dissemination, facilitators of its future applications, and opportunities to broaden its equitable access.

We therefore sought to analyze patterns, correlates, and clinical outcomes associated with implementation of physician telemedicine visits during treatment for a cohort of consecutive patients treated in our large, academic radiation oncology practice upon first implementation, activated by the COVID‐19 pandemic. Our objectives were to (1) identify temporal trends in telemedicine use, benchmarked by key policy changes over the study period, (2) identify variations in telemedicine uptake and assess correlations with patient and physician characteristics, and (3) examine whether the use of telemedicine versus in‐person care increased the risks of acute adverse clinical outcomes, including treatment toxicity, emergency care use, and 30‐day hospitalization rates.

## 2 METHODS

This retrospective cohort study (protocol # PA19‐0352) was approved by the institutional review board, and the need for informed consent was waived.^23^

### 2.1 Study sample

We identified 468 consecutive complete RT courses in 461 unique patients at our institution from March 16 through June 1, 2020. For the seven patients who had more than one course during the study period, these separate courses were considered independent analytic units, as they were, for example, separated by time or managed by different physicians. Each unit for analysis was considered an independent event in the primary analysis. In a sensitivity analysis, only the first event for the 461 unique patients was included. Patients with any cancer disease site, stage, treatment intent (palliative or curative), and treatment site who were treated at the main academic center or four regional community satellite practices were included. The start date of the study reflected the date of the institution's implementation of an interactive audiovisual visit telemedicine platform for managing radiation oncology clinical care visits.^24^

All patients had one or more weekly management visits during their course of RT, during which a board‐certified radiation oncologist evaluated the patient. Information documented during weekly management visits included, as applicable, grading of toxicities during treatment according to Radiation Therapy Oncology Group (RTOG)^25^ and Common Terminology Criteria for Adverse Events (CTCAE) v4.0^26^ criteria and formulation of an overall assessment and plan including management of clinical toxicities.

### 2.2 Covariates

Patient covariates included demographic, insurance, disease, and treatment characteristics and were abstracted from the electronic medical records. Race and ethnicity were based on patients' self‐reported race and ethnicity in their electronic medical record in accordance with guidelines set forth in the 11th edition of the American Medical Association Manual of Style.^27^ Distance between treatment facility and home was calculated based on patients' reported home address. Physician demographic covariates, including self‐reported race and ethnicity, were obtained from publicly available data from the state Medical Board.^28^

### 2.3 Outcomes

For the first and second study objective, the outcome was use of telemedicine, dichotomized as use versus no use, during the treatment course. Patients were considered to have used telemedicine if they had had at least one weekly visit with a physician during the entire course of treatment, as opposed to not using telemedicine but having all weekly physician visits in‐person. For the third objective, the outcomes included: (1) occurrence and grade of CTCAE physician‐graded toxicities during the active RT treatment delivery period; (2) emergency room visit within 30 days of the last date of RT in the course; and (3) unplanned hospitalization within 30 days of the last date of RT (elective hospitalizations, e.g., for a planned curative tumor resection, were excluded from this event definition).

### 2.4 Statistical analysis

The Cochran‐Armitage test was used to analyze temporal trends in telemedicine use. Univariable associations of patient‐level and physician‐level covariates with telemedicine use were tested with the chi‐squared test for categorical covariates and Wilcoxon rank‐sum or Kruskal–Wallis tests for continuous variables. A multilevel multivariable logistic model using random intercept, with patients (level 1) clustered within physicians (level 2), identified the association of patient‐ and physician‐level characteristics with telemedicine use. A residual intraclass correlation coefficient was calculated to evaluate the strength of the cluster of relationships to characterize the impact of physician‐level variation on outcomes. For this multivariate model, patient race and ethnicity were categorized as White non‐Hispanic versus Black non‐Hispanic or any Hispanic ethnicity versus all other based on univariate distributions. Based on univariate distributions and collapsing categories in which the absolute count of physicians was <10 in a category (to ensure anonymity), physician race, and ethnicity were categorized as White non‐Hispanic versus Asian versus all other.

Univariate CTCAE toxicities during RT, 30‐day emergency visits, and 30‐day unplanned hospitalization frequencies by telemedicine use category were compared using the chi‐squared test and multivariable associations using logistic models. *p* values were two‐sided. The threshold of 0.05 was used to determine statistical significance. Statistical analyses were performed using SAS v. 9.4 and Stata Version 16.0 (StataCorp).

## 3 RESULTS

### 3.1 Uptake

**FIGURE 1:**
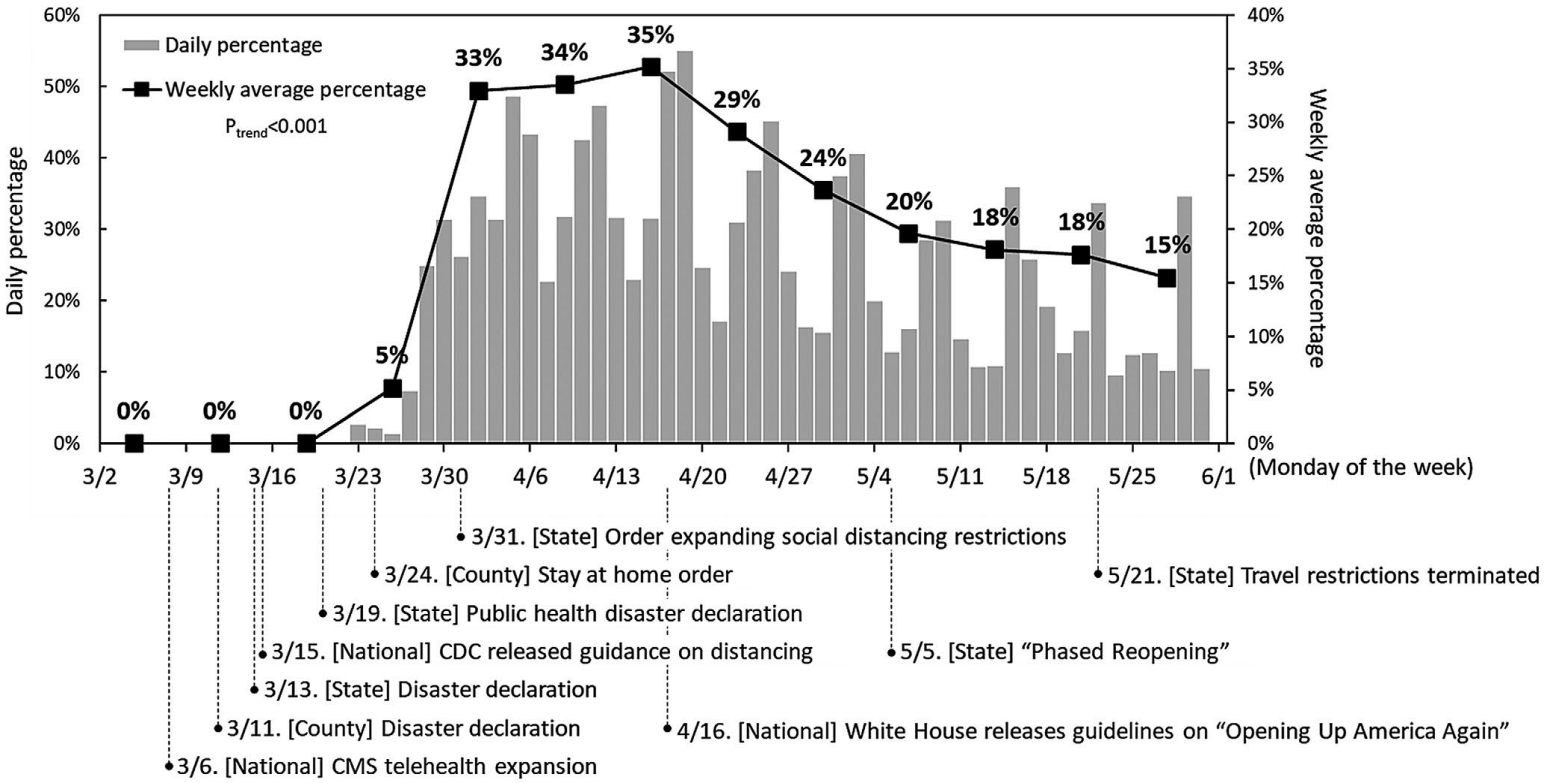
Telemedicine use daily and as a proportion of total weekly physician visits, with key national, state, and local policy events during the study period

Telemedicine was used as a component of 33% of patient treatment courses (*n* = 155 of 468) whereas in‐person only oncologist management was used in 67% (*n* = 313). The rolling proportion of weekly clinical appointment visits where telemedicine was used (of the total number of visits by week) over time is displayed in Figure 1. Significant temporal changes were noted in telemedicine use (*p*_trend_ < 0.001). Initial implementation across the oncology care center occurred after being enabled by the Centers for Medicare & Medicaid Services (CMS) rule on March 6, 2020 for temporary payment for telemedicine services with the same amount as for in‐person care, followed by national expansion under the Coronavirus Preparedness and Response Supplemental Appropriations Act. Use of telemedicine rose rapidly after its initial implementation, correlating temporally with the issuances of a county stay‐at‐home order and state social‐distancing restrictions, county and state disaster declarations, and national Centers for Disease Control and Prevention (CDC) distancing guidelines. Telemedicine use peaked at 35% of total visits in the fourth week after implementation. After this, the telemedicine visit proportion declined, coinciding with release of US national guidelines from the White House, “Opening Up America Again,” on April 16, 2020.^29^ Telemedicine use was at its nadir, 15%, during the last week of the study period.

### 3.2 Patient and physician characteristics

Patient, disease, treatment, and treating physician characteristics are shown in Table S1. Median patient age at treatment was 64 years (interquartile range [IQR] 52–72 years). Patient sex was approximately evenly split between male and female. The most commonly represented patient races/ethnicities were non‐Hispanic White (71%), any Hispanic ethnicity (13%), and non‐Hispanic Black (9%). The most commonly treated primary disease sites were lung and thorax (17%), breast (16%), and gastrointestinal (15%). Most patients were treated with non‐palliative intent. Nearly half of patients received five or fewer daily treatments (RT “fractions”). Most patients (52%) reported a home address within 75 miles of their treatment facility. Of 64 treating physicians, median age was 45 years (IQR 42–53 years); 53% were male; 41% were white non‐Hispanic, and 31% were Asian. On a per‐physician basis, the median proportion of each physician's visits managed by telemedicine was 18% (range 0%–100%; IQR 0%–38%).

### 3.3 Multilevel correlates of telemedicine use

Univariate patient‐level correlates of telemedicine use are displayed in Table 1. No differences in use were found by patient age, sex, or distance to treatment facility. When frequency of telemedicine use was compared for White non‐Hispanic patients versus others, White non‐Hispanics were more likely to have used telemedicine than non‐White patients (*p* = 0.04). Use of telemedicine also varied by disease site (*p* < 0.001), with patients with lung or other thoracic cancers, gastrointestinal cancers, or head and neck cancers most likely to use telemedicine. Patients treated with longer radiation courses were also more likely to use telemedicine (*p* = 0.006).

**TABLE 1:** Patient characteristics correlated with use of telemedicine for weekly physician management visits

Characteristic	Using telemedicine *N* = 155	Not using telemedicine *N* = 313	*p*‐value
Age (years)			
Median (IQR)	64 (53, 71)	64 (52, 72)	0.66
Sex			
Female	71 (46%)	168 (54%)	0.11
Male	84 (54%)	145 (46%)	
Race and ethnicity			
White non‐Hispanic	120 (77%)	213 (68%)	0.17
Black non‐Hispanic	11 (7%)	33 (11%)	
Any Hispanic	14 (9%)	45 (14%)	
Other	10 (6%)	22 (7%)	
Primary insurance			
Non‐Medicare	88	172	0.09
Medicare	67	141	
Distance to treatment facility			
<25 miles	43 (28%)	101 (32%)	0.28
25–50 miles	22 (14%)	55 (18%)	
50–75 miles	7 (5%)	13 (4%)	
75–150 miles	18 (12%)	33 (11%)	
150–500 miles	41 (26%)	58 (19%)	
500–5000 miles	22 (14%)	40 (13%)	
International	2 (1%)	13 (4%)	
Primary cancer site			
Breast	12 (8%)	65 (21%)	**<0.001**
Central nervous system	8 (5%)	3 (1%)	
Connective and soft tissue	2 (1%)	16 (5%)	
Esophagus	3 (2%)	2 (1%)	
Other gastrointestinal	24 (15%)	44 (14%)	
Genitourinary	15 (10%)	42 (13%)	
Gynecologic	4 (3%)	8 (2%)	
Head and neck	24 (15%)	26 (8%)	
Hematologic	11 (7%)	40 (13%)	
Lung and thorax	40 (26%)	40 (13%)	
Skin	4 (3%)	12 (4%)	
Other^a^	8 (5%)	15 (5%)	
Treatment for disease recurrence			
No	133 (86%)	265 (85%)	0.74
Yes	22 (14%)	48 (15%)	
Treatment goal			
Non‐Palliative	102 (66%)	184 (59%)	0.14
Palliative	53 (34%)	129 (41%)	
Number of radiotherapy fractions			
1–5	60 (39%)	164 (52%)	**0.005**
6+	95 (61%)	149 (48%)	

Abbreviations: IQR, interquartile range.

^a^ Includes adrenal gland, bone, neuroendocrine, non‐cancer, retroperitoneum and peritoneum, and unspecified.

Significant p‐values are highlighted in bold.

The multivariable multilevel model identifying patient and physician characteristics associated with telemedicine use is shown in Table 2. The patient‐level demographic factors of age, sex, primary insurance payer, and distance to treatment facility were not significantly associated with telemedicine use. However, non‐Hispanic White patients remained more likely than Black or Hispanic patients to use telemedicine (odds ratio [OR] = 2.20, 95% confidence interval [CI] 1.03–4.72, *p* = 0.04). Patients undergoing longer‐duration courses (≥6 fractions) also remained more likely than those treated with 1–5 fractions to use telemedicine (OR = 4.49, 95% CI 2.29–8.80, *p* < 0.001). Differences in telemedicine use by disease site were not significant after covariate adjustment.

**TABLE 2:** Multilevel model for patient and physician characteristics associated with use of cancer telemedicine visits

	OR	95% CI	*p*‐value
Patient level			
Age	1.00	0.97–1.02	0.69
Sex			
Female	(Reference)		
Male	1.25	0.63–2.47	0.52
Race and ethnicity			
White non‐Hispanic	(Reference)		
Black or Hispanic	0.46	0.21–0.98	**0.04**
Other	0.66	0.20–2.14	0.48
Primary insurance			
Non‐medicare	(Reference)		
Medicare	0.72	0.32–1.63	0.42
Distance to treatment facility			
<25 miles	(Reference)		
25–50 miles	0.68	0.28–1.67	0.39
50–75 miles	1.58	0.31–8.04	0.58
75–150 miles	0.70	0.26–1.91	0.48
150–500 miles	1.19	0.53–2.64	0.67
500–5000 miles	0.63	0.24–1.62	0.34
International	0.12	0.01–1.06	0.06
Primary cancer site			0.14
Lung and Thorax	(Reference)		
Breast	0.64	0.13–3.32	0.59
Central nervous system	5.33	0.53–53.79	0.15
Connective and soft tissue	0.57	0.06–5.52	0.63
Esophagus	0.43	0.04–4.92	0.49
Other gastrointestinal	1.71	0.41–7.15	0.46
Genitourinary	1.24	0.28–5.44	0.78
Gynecologic	3.25	0.40–26.65	0.27
Head and neck	5.89	1.34–25.80	**0.02**
Hematologic	6.15	0.77–49.00	0.09
Skin	2.88	0.40–20.78	0.29
Other^a^	0.76	0.15–3.77	0.73
Treatment for disease recurrence			
No	(Reference)		
Yes	0.53	0.23–1.24	0.14
Treatment goal			
Non‐palliative	(Reference)		
Palliative	0.76	0.40–1.45	0.40
Number of radiotherapy fractions			
1–5	(Reference)		
6+	4.49	2.29–8.80	<**0.001**
Physician level			
Age	1.02	0.94–1.10	0.68
Sex			
Female	(Reference)		
Male	1.89	0.51–7.07	0.34
Race and ethnicity			
White non‐Hispanic	(Reference)		
Asian	7.50	1.58–35.56	**0.01**
Other^b^	0.84	0.16–4.47	0.83

Abbreviations: CI, confidence interval; OR, odds ratio.

^a^ Includes adrenal gland, bone, neuroendocrine, non‐cancer, retroperitoneum and peritoneum, and unspecified.

^b^ Includes ethnicity or race categories with counts <10.

Significant p‐values are highlighted in bold.

The model intraclass correlation coefficient demonstrated that 43% of the variation in use was physician‐level driven. There was significant variation in the patterns of telemedicine use by physician (*p* = 0.0005). However, of the known physician‐level characteristics, neither physician age nor sex were associated with telemedicine use. Even after adjusting for patient and physician level covariates, Asian physicians were more likely (OR = 7.50, 95% CI 1.58–35.56, *p* = 0.01) than White non‐Hispanic physicians to use a telemedicine visit. In a sensitivity analysis, when the multilevel modeling included the 461 unique patients undergoing first courses of RT within the study period, results remained similar. Specifically, non‐Hispanic White patient race/ethnicity (OR = 2.15, 95% CI 1.00–4.61, *p* = 0.05), patients undergoing ≥6 fractions of RT (OR = 4.50, 95% CI 2.29–8.86, *p* < 0.0001), and Asian physician race/ethnicity (OR = 7.05, 95% CI 1.49–33.43, *p* = 0.01) remained associated with telemedicine use.

### 3.4 Clinical outcomes: Toxicities and adverse events

No differences were found in the frequency of acute RT toxicities, patient‐reported pain, emergency room visits within 30 days, or unplanned hospitalizations within 30 days for patients who were managed using any telemedicine versus patients managed using in‐person physician visits only (Table 3). In multivariable logistic models, telemedicine use was not a predictor of the combined outcome of any emergency room visit or unplanned hospitalization within 30 days (OR = 1.47, 95% CI 0.73–2.98, *p* = 0.28). The only significant predictor of emergency visit or unplanned hospitalization was treatment with palliative intent (Table S2).

**TABLE 3:** Frequency of adverse events by telemedicine use

	Using telemedicine *N* = 155	Not using telemedicine *N* = 313	*p*‐value
Acute radiotherapy toxicity^a^			
Grade 1	108 (70%)	208 (66%)	0.48
Grade 2	44 (28%)	70 (22%)	0.15
Grade 3	4 (3%)	8 (3%)	0.99
Emergency room visit^b^	18 (12%)	23 (7%)	0.12
Unplanned hospitalization^b^	16 (10%)	21 (7%)	0.17

^a^ Per Common Terminology Criteria for Adverse Events (CTCAE) v4.0 and Radiation Therapy Oncology Group (RTOG) toxicity grading for acute radiation effects.

^b^ Within 30 days of radiotherapy.

## 4 DISCUSSION

In this cohort of cancer patients, we identified that multilevel factors impacted early uptake and use of oncology telemedicine for weekly physician visits for radiation oncology care. Temporal patterns of initial uptake and eventual decrease in telemedicine use followed benchmark local, state, and national policies influencing health insurance payment for telehealth‐based care as well as COVID‐19 disease mitigation and management. At the patient level, the only clinical factor that remained a statistically significant correlate of telemedicine use was length of treatment. Longer treatment courses, which by definition required more weekly physician visits, likely offered more opportunities for patients and physician to opt for at least one telemedicine‐based visit. The sole patient demographic factor that remained a correlate of a higher likelihood of telemedicine use was non‐Hispanic White race/ethnicity, compared with significantly lower uptake among Black and Hispanic patients. Non‐Hispanic White physicians, however, were less likely to use telemedicine.

Notably, patients who used telemedicine did not demonstrate a statistically significant increase in the frequency acute treatment‐related toxicities, emergency room visits, or unplanned hospitalizations. Despite initial concern that oncology telemedicine might result in lower quality care,^30^ these early outcomes data provide initial reassurance of telemedicine's potential for effectiveness as a modality to support high‐quality cancer care delivery, although evaluation of long‐term patient outcomes is needed. Evolving iterations of oncology telemedicine practices may continue to strengthen the clinical outcomes achievable with this approach by improving the incorporation of patient‐reported outcomes, wearable data, and deeper user engagement, such as coaching support to improve use of and comfort with the necessary technology.^31^, ^32^ Provision of high‐quality care may also be facilitated by the development of expert guidelines regarding how to select appropriate patients for telemedicine, such as those described in the recently published American Society of Clinical Oncology Standards and Practice Recommendations.^33^

It is not known whether the observed differences in this study in racial and ethnic patient‐ and physician‐level variations in use represent disparities in care delivery, that is, a disadvantage in the care for minority patients. Variations could represent intentional patient preferences or needs. Unlike quality benchmarked practices in oncology such as use of guideline‐concordant care, the optimal selection criteria for telemedicine care in cancer patients are still undefined. For example, a survey study of 56 cancer patients showed that 88% were satisfied with virtual visits overall, but 27% of patients receiving radiation were dissatisfied with virtual on‐treatment visits, a proportion significantly greater than patients dissatisfied by virtual new patient visits (6%) or follow‐up visits (0%).^11^ A pilot study of a telemedicine platform for the first appointment after RT completion revealed 86% patient satisfaction and 82% physician confidence in assessments of treatment‐related toxicity (though less so for skin toxicity specific to breast radiation).^34^ A survey of radiation oncologists reported that 71% saw at least no difference in their ability to treat cancer appropriately via telemedicine visits versus in‐person visits, with 16% reporting potential improvement in overall visit quality with telemedicine. Nevertheless, the conclusion in that study was that 5%–30% of cancer patients may still be better suited for in‐person physician evaluations to optimize treatment recommendations and planning.^6^ Findings from a survey by the National Comprehensive Cancer Network Electronic Health Record Oncology Advisory Group demonstrate provider confidence that a substantial fraction of visits for cancer patients could be effectively and safely conducted using telemedicine.^16^ Although patients and physicians have reported the belief that telemedicine could increase safety and convenience during the COVID‐19 pandemic, authors have also raised the consideration that certain subgroups, such as patients with impaired senses or cognition, those for whom physical examination could substantially alter management, those for whom sensitive discussions are needed, and those without access to smartphones and broadband internet may nevertheless prefer in‐person visits.^35^, ^36^, ^37^, ^38^ Still unknown, however, is how social and demographic patient characteristics may modify an individual's comparative benefit from virtual versus in‐person visits. Given our study's findings that telemedicine use in oncology could vary by key factors such as race, additional studies on variations in patient‐reported benefits of telemedicine by sociodemographic factors may be needed.

Inequity as a possible contributor to racial and ethnic variation in telemedicine use in this study cannot be ignored. Racial, ethnic, and socioeconomic disparities continue to have adverse effects in cancer care, despite considerable research on variation in quality of care, policy changes to promote access to care, and ongoing efforts to improve cultural competence of the workforce.^39^ These early findings prompt the question whether rapid implementation of telemedicine in the COVID‐19 setting may reflect the socioeconomic inequalities in health underscored by the pandemic in the United States.^40^ A recent analysis of claims data from a large commercial insurer examined patients with newly diagnosed cancer at the start of the pandemic. This study showed that patients in the highest quartile of a socioeconomic index were 31% more likely to utilize telemedicine when compared with those in the lowest quartile.^41^ Although telemedicine is a novel potential pathway for accessing cancer care, it is not universally accessible, and patient factors such as literacy, familiarity with technology, or access to resources such as mobile devices and wireless connections may represent barriers to telemedicine access in disadvantaged populations.^42^ Our finding that 43% of the variation in telemedicine use patterns was driven by the physicians also emphasizes the importance of engaging providers, care teams, and health systems in understanding and directly addressing potential inequities in telemedicine access.^43^, ^44^ In the current study, Asian race among physicians was strongly associated with telemedicine use, even after adjustment for physician factors, such as age and gender, and adjustment for factors clustered by physician, such as disease site specialty and treatment facility location. The underlying mechanism for racial differences by physician in use of telemedicine result is unclear. Possible physician‐level characteristics that may need to be explored in future studies include other detailed confounding factors such as training and technological expertise. Because factors underlying racial variation in physician uptake of telemedicine have not been defined to date, barriers and facilitators of telemedicine use from the oncologist perspective also need to be identified.

Lower use of audiovisual telemedicine visits was not identified among older patients in our sample compared with younger patients. A recent study by Stevens et al. of a general patient sample showed that older patients were more likely to use telehealth in visits during the early COVID‐19 pandemic, though in that study, they were less likely to use video technology than younger patients.^45^ Caregivers are frequently present for cancer patients and may be an important support for helping older patients to use telemedicine.

This study has limitations to consider. Analysis of the multilevel correlates of use in radiation oncology may not be directly translatable to other types of oncology care such as surgery, chemotherapy, or stem cell transplants and will require additional validation in these other settings. Patient income and education were not available covariates in this retrospective analysis, and these variables may need additional investigation as potential mediators of racial/ethnic differences in use. Emergency visits and unplanned hospitalizations outside the authors' institution and its affiliated practices were not captured, which may have resulted in underestimation of these events, and thus additional validation of clinical outcomes after telemedicine use, including longer‐term outcomes, is needed. In addition, results of the current study are not intended to guide the prospective selection of patients best suited for telemedicine in an oncology setting. Physician characteristics available in this study were relatively limited. Additional studies exploring such detailed physician‐level data are needed to better understand mechanisms of physician telemedicine practice patterns in the oncology setting. Moreover, multi‐site analyses will be needed in order to include a wider spectrum of physicians and physician practice patterns and validate findings.

## 5 CONCLUSION

We found that patient, physician, and policy factors influenced uptake and use of oncology telemedicine in a radiation oncology care delivery setting, but patterns of use were relatively highly physician‐driven. Given a lack of clinical outcomes data to date on use of telemedicine for cancer care, the results of this study are reassuring in demonstrating that telemedicine use for weekly physician management visits was associated with similar profiles of acute clinical toxicities and adverse outcomes to those seen for physician management delivered solely in‐person. Nevertheless, given the lower uptake of telemedicine among non‐White patients, additional study is needed to characterize overarching patterns of variation in oncology telemedicine access across cancer populations, especially medically underserved and socioeconomically vulnerable groups. Continuing implementation strategies should address the influential multilevel predictors of telemedicine use to optimize the potential for this technology to surmount—and not exacerbate—barriers to quality cancer care.

## Funding information

This work was supported in part by funding from P30 CA016672 from the National Cancer Institute, National Institutes of Health (MD Anderson Cancer Center). B.D. is supported by the RSNA Research & Education Foundation Grant RR2111. G.L.S. is supported by the National Cancer Institute (NIH/NCI K07CA211804). B.D.S. is supported by the Cancer Prevention and Research Institute of Texas Grant RP160674 and the National Cancer Institute Grants R01 CA207216 and R01 1CA225646.

## CONFLICT OF INTEREST

BD reports consulting honoraria from Sermo, Inc. BDS reports a royalty and equity interest in Oncora Medical. PD reports consulting/advisory relationships with American Society for Radiation Oncology and National Cancer Institute. AC reports a consulting relationship with Mathematica.

## AUTHOR CONTRIBUTIONS

Conception or design of the work: G. Smith. Data collection: Fu, Chen, G. Smith. Data analysis and interpretation: All authors. Drafting the article: De, G. Smith. Critical revision of the article: All authors. Final approval of the version to be published: All authors.

## Supporting information


Table S1
Table S2

## Data Availability

Data for this study are not available.

## References

[1] Robilotti EV , Babady NE , Mead PA , et al. Determinants of COVID‐19 disease severity in patients with cancer. Nat Med. 2020;26:1218‐1223. 32581323 10.1038/s41591-020-0979-0 PMC7785283

[2] Alom S , Chiu CM , Jha A , Lai SHD , Yau THL , Harky A . The effects of COVID‐19 on cancer care provision: a systematic review. Cancer Control. 2021;28:1073274821997425. 33631953 10.1177/1073274821997425 PMC8482720

[3] Maroongroge S , Smith B , Bloom ES , et al. Telemedicine for radiation oncology in a post‐COVID world. Int J Radiat Oncol Biol Phys. 2020;108:407‐410. 32890522 10.1016/j.ijrobp.2020.06.040 PMC7462809

[4] Lafata JE , Smith AB , Wood WA , Fitzpatrick B , Royce TJ . Virtual visits in oncology: enhancing care quality while designing for equity. JCO Oncol Pract. 2021;17:220‐223. 33539180 10.1200/OP.20.00645

[5] Royce TJ , Sanoff HK , Rewari A . Telemedicine for cancer care in the time of COVID‐19. JAMA Oncol. 2020;6:1698‐1699. 32672821 10.1001/jamaoncol.2020.2684 PMC11021845

[6] Zhang H , Cha EE , Lynch K , et al. Radiation oncologist perceptions of telemedicine from consultation to treatment planning: a mixed‐methods study. Int J Radiat Oncol Biol Phys. 2020;108:421‐429. 32890525 10.1016/j.ijrobp.2020.07.007 PMC7462757

[7] Wakefield DV , Sanders T , Wilson E , et al. Initial impact and operational responses to the COVID‐19 pandemic by American radiation oncology practices. Int J Radn Oncol Biol Phys. 2020;108:356‐361. 10.1016/j.ijrobp.2020.06.060 PMC7462779 32890512

[8] Lewis GD , Hatch SS , Wiederhold LR , Swanson TA . Long‐term institutional experience with telemedicine services for radiation oncology: a potential model for long‐term utilization. Adv Radiat Oncol. 2020;5:780‐782. 32391444 10.1016/j.adro.2020.04.018 PMC7205717

[9] Orazem M , Oblak I , Spanic T , Ratosa I . Telemedicine in radiation oncology post–COVID‐19 pandemic: there is no turning back. Int J Radn Oncol Biol Phys. 2020;108:411‐415. 10.1016/j.ijrobp.2020.06.052 PMC7462837 32890523

[10] Shaverdian N , Gillespie EF , Cha E , et al. Impact of telemedicine on patient satisfaction and perceptions of care quality in radiation. Oncology. 2021;1:1‐7. 10.6004/jnccn.2020.7687 PMC8254817 33395627

[11] Gutkin PM , Prionas ND , Minneci MO , et al. Telemedicine in radiation oncology: is it here to stay? Impacts on patient care and resident education. Int J Radn Oncol Biol Phys. 2020;108:416‐420. 10.1016/j.ijrobp.2020.06.047 PMC7462793 32890524

[12] Heyer A , Granberg RE , Rising KL , Binder AF , Gentsch AT , Handley NR . Medical oncology professionals' perceptions of telehealth video visits. JAMA Netw Open. 2021;4:e2033967. 33443581 10.1001/jamanetworkopen.2020.33967 PMC7809588

[13] Granberg RE , Heyer A , Rising KL , Handley NR , Gentsch AT , Binder AF . Medical oncology patient perceptions of telehealth video visits. 0: OP.21.00086. 10.1200/OP.21.00086 34288697

[14] Meno M , Abe J , Fukui J , Braun‐Inglis C , Pagano I , Acoba J . Telehealth amid the COVID‐19 pandemic: perception among Asian, Native Hawaiian and Pacific Islander cancer patients. Future Oncol. 2021;17:3077‐3085. 34102878 10.2217/fon-2021-0136 PMC8202507

[15] Fassas S , Cummings E , Sykes KJ , Bur AM , Shnayder Y , Kakarala K . Telemedicine for head and neck cancer surveillance in the COVID‐19 Era: promise and pitfalls. Head Neck. 2021;43:1872‐1880. 33660409 10.1002/hed.26659 PMC8013462

[16] Tevaarwerk AJ , Chandereng T , Osterman T , et al. Oncologist perspectives on telemedicine for patients with cancer: a national comprehensive cancer network survey. JCO Oncol Pract. 2021;17:e1318‐e1326. 34264741 10.1200/OP.21.00195 PMC9810123

[17] Liu R , Sundaresan T , Reed ME , Trosman JR , Weldon CB , Kolevska T . Telehealth in oncology during the COVID‐19 outbreak: bringing the house call back virtually. Am Soc Clin Oncol. 2020;16:289‐293. 10.1200/OP.20.00199 32364826

[18] Lynch JA , Berse B , Rabb M , et al. Underutilization and disparities in access to EGFR testing among Medicare patients with lung cancer from 2010–2013. BMC Cancer. 2018;18:306. 29554880 10.1186/s12885-018-4190-3 PMC5859516

[19] De B , Kaiser KW , Ludmir EB , et al. Radiotherapy clinical trial enrollment during the COVID‐19 pandemic. Acta Oncol. 2020;60:312‐315. 33356801 10.1080/0284186X.2020.1865564

[20] Spratt DE , Chan T , Waldron L , et al. Racial/ethnic disparities in genomic sequencing. JAMA Oncol. 2016;2:1070‐1074. 27366979 10.1001/jamaoncol.2016.1854 PMC5123755

[21] Aizer AA , Wilhite TJ , Chen MH , et al. Lack of reduction in racial disparities in cancer‐specific mortality over a 20‐year period. Cancer. 2014;120:1532‐1539. 24863392 10.1002/cncr.28617

[22] LeMasters T , Madhavan SS , Sambamoorthi U , Hazard‐Jenkins HW , Kelly KM , Long D . Receipt of guideline‐concordant care among older women with stage I–III breast cancer: a population‐based study. J Natl Compr Canc Netw. 2018;16:703‐710. 29891521 10.6004/jnccn.2018.7004 PMC6091512

[23] The World Medical Association , Ferney‐Voltaire F . Ethical principles for medical research involving human subjects. Declaration of Helsinki; 2013. 10.1001/jama.2013.281053 24141714

[24] Noticewala SS , Koong AC , Bloom ES , et al. Radiation oncology strategies to flatten the curve during the coronavirus disease 2019 (COVID‐19) pandemic: experience from a large tertiary cancer center. Adv Radiat Oncol. 2020;5:567‐572. 32775771 10.1016/j.adro.2020.04.038 PMC7240274

[25] Cox JD , Stetz J , Pajak TF . Toxicity criteria of the radiation therapy oncology group (RTOG) and the European Organization for Research and Treatment of cancer (EORTC). Int J Radiat Oncol Biol Phys. 1995;31:1341‐1346. 7713792 10.1016/0360-3016(95)00060-C

[26] U.S. Department of Health and Human Services . Common terminology criteria for adverse events (CTCAE) Version 4.0. U.S. Department of Health and Human Services; 2010. https://ctep.cancer.gov/protocoldevelopment/electronic_applications/ctc.htm

[27] Christiansen S , Iverson C , Flanagin A , et al. AMA Manual of Style: A Guide for Authors and Editors. 11th ed. Oxford University Press; 2020. Accessed November 20, 2021. https://www.amamanualofstyle.com/

[28] Healthcare Provider Search Results. https://profile.tmb.state.tx.us/ (accessed 2021).

[29] The Whitehouse, Centers for Disease Control and Prevention. Guidelines: Opening Up America Again (2020).

[30] Uscher‐Pines L , Sousa J , Jones M , et al. Telehealth use among safety‐net organizations in California during the COVID‐19 pandemic. JAMA. 2021;325:1106‐1107. 33528494 10.1001/jama.2021.0282 PMC7856569

[31] Akhu‐Zaheya LM , Waed YS . The effect of short message system (SMS) reminder on adherence to a healthy diet, medication, and cessation of smoking among adult patients with cardiovascular diseases. Int J Med Inform. 2017;98:65‐75. 28034414 10.1016/j.ijmedinf.2016.12.003

[32] Weinstein RS , Lopez AM , Joseph BA , et al. Telemedicine, telehealth, and mobile health applications that work: opportunities and barriers. Am J Med. 2014;127:183‐187. 24384059 10.1016/j.amjmed.2013.09.032

[33] Zon RT , Kennedy EB , Adelson K , et al. Telehealth in oncology: ASCO standards and practice recommendations. JCO Oncol Pract. 2021;17:546‐564. 34319760 10.1200/OP.21.00438

[34] Miller RC , Simone B , Nowak Choi KA , et al. A pilot trial using telehealth in radiation oncology: the future of healthcare is virtual. Int J Radiat Oncol Biol Phys. 2020;108:S53.

[35] Kitamura C , Zurawel‐Balaura L , Wong RK . How effective is video consultation in clinical oncology? A systematic review. Curr Oncol. 2010;17:17‐27. 10.3747/co.v17i3.513 PMC2880899 20567623

[36] Daggubati LC , Eichberg DG , Ivan ME , et al. Telemedicine for outpatient neurosurgical oncology care: lessons learned for the future during the COVID‐19 pandemic. World Neurosurg. 2020;139:e859‐e863. 32450309 10.1016/j.wneu.2020.05.140 PMC7243783

[37] Wolf I , Waissengrin B , Pelles S . Breaking bad news via telemedicine: a new challenge at times of an epidemic. Oncologist. 2020;25:e879‐e880. 32304624 10.1634/theoncologist.2020-0284 PMC7288637

[38] Holstead RG , Robinson AG . Discussing serious news remotely: navigating difficult conversations during a pandemic. JCO Oncol Pract. 2020;16:363‐368. 32421390 10.1200/OP.20.00269

[39] Moy B , Polite BN , Halpern MT , et al. American Society of Clinical Oncology policy statement: opportunities in the patient protection and affordable care act to reduce cancer care disparities. J Clin Oncol. 2011;29:3816‐3824. 21810680 10.1200/JCO.2011.35.8903

[40] Schmidt AL , Bakouny Z , Bhalla S , et al. Cancer care disparities during the COVID‐19 pandemic: COVID‐19 and cancer outcomes study. Cancer Cell. 2020;38:769‐770. 33176161 10.1016/j.ccell.2020.10.023 PMC7609043

[41] Katz AJ , Haynes K , Du S , Barron J , Kubik R , Chen RC . Evaluation of telemedicine use among US patients with newly diagnosed cancer by socioeconomic status. JAMA Oncol. 2021;8:161. 10.1001/jamaoncol.2021.5784 PMC8603229 34792526

[42] Franco I , Perni S , Wiley S , Drapek L . Equity in radiation oncology post‐COVID: bridging the telemedicine gap. Int J Radiat Oncol Biol Phys. 2020;108:479‐482. 32890538 10.1016/j.ijrobp.2020.06.051 PMC7462878

[43] Medicine Io . Unequal Treatment: Confronting Racial and Ethnic Disparities in Health Care. The National Academies Press; 2003. 25032386

[44] Abdel‐Wahab M , Rosenblatt E , Prajogi B , Zubizarretta E . Opportunities in telemedicine, lessons learned after COVID‐19 and the way into the future. Int J Radiat Oncol Biol Phys. 2020;108:438‐443. 32890528 10.1016/j.ijrobp.2020.07.006 PMC7462967

[45] Stevens JP , Mechanic O , Markson L , O'Donoghue A , Kimball AB . Telehealth use by age and race at a single academic medical center during the COVID‐19 pandemic: retrospective cohort study. J Med Internet Res. 2021;23:e23905. 33974549 10.2196/23905 PMC8139390

