# Supplementary material for: Patient, physician, and policy factors underlying variation in use of telemedicine for radiation oncology cancer care
Source: Cancer Med. 2022 Mar 16;11(10):2096–105. doi: 10.1002/cam4.4555 (PMC9119354; doi:10.1002/cam4.4555)
Supplement: Supplementary file 1 — Table S1 Table S2 [file CAM4-11-2096-s001.docx]

**Table S1**. Patient, disease, treatment and physician characteristics

| *Patient Characteristics* | No. | % |
| --- | --- | --- |
| Age, median (IQR) | 64 (52, 72) |  |
| Sex |  |  |
| Female | 239 | 51.1 |
| Male | 229 | 48.9 |
| Race and ethnicity |  |  |
| White non-Hispanic | 333 | 71.2 |
| Black non-Hispanic | 44 | 9.4 |
| Hispanic | 59 | 12.6 |
| Other | 32 | 6.8 |
| Primary insurance |  |  |
| Non-Medicare | 260 | 55.6 |
| Medicare | 208 | 44.4 |
| Distance to treatment facility |  |  |
| < 25 miles | 144 | 30.8 |
| 25-50 miles | 77 | 16.5 |
| 50-75 miles | 20 | 4.3 |
| 75-150 miles | 51 | 10.9 |
| 150-500 miles | 99 | 21.2 |
| 500-5000 miles | 62 | 13.3 |
| International | 15 | 3.2 |
| Primary cancer site |  |  |
| Breast | 77 | 16.5 |
| Central Nervous System | 11 | 2.4 |
| Connective and soft tissue | 18 | 3.9 |
| Esophagus | 5 | 1.1 |
| Gastrointestinal | 68 | 14.5 |
| Genitourinary | 57 | 12.2 |
| Gynecology | 12 | 2.6 |
| Head and Neck | 50 | 10.7 |
| Hematology | 51 | 10.9 |
| Lung and Thorax | 80 | 17.1 |
| Skin | 16 | 3.4 |
| Other (adrenal gland, bone, neuroendocrine, non-cancer, retroperitoneum and peritoneum and unspecified) | 23 | 4.9 |
| Treatment for disease recurrence |  |  |
| No | 398 | 85.0 |
| Yes | 70 | 15.0 |
| Treatment goal |  |  |
| Non-palliative | 286 | 61.1 |
| Palliative | 182 | 38.9 |
| Number of radiotherapy fractions |  |  |
| 1-5 | 224 | 47.9 |
| 6-10 | 83 | 17.7 |
| 11-24 | 88 | 18.8 |
| 25+ | 73 | 15.6 |
|  | | |
| *Physician Characteristics* |  |  |
| Age, median (IQR) | 64 (42, 53) |  |
| Sex |  |  |
| Female | 30 | 46.9 |
| Male | 34 | 53.1 |
| Race/ethnicity |  |  |
| White non-Hispanic | 26 | 40.6 |
| Asian | 20 | 31.3 |
| Other^†^ | 18 | 28.1 |

^†^*Includes ethnicity or race categories with counts less than 10*

**Table S2.** Multivariable model for patient characteristics associated with emergency visit or unplanned hospitalization

|  | OR | 95% CI | P-value |
| --- | --- | --- | --- |
| *Patient Level* |  |  |  |
| Use of telemedicine |  |  |  |
| None | (Reference) |  |  |
| Any use | 1.47 | 0.73-2.98 | 0.28 |
| Age | 1.01 | 0.98-1.04 | 0.54 |
| Sex |  |  |  |
| Female | (Reference) |  |  |
| Male | 0.66 | 0.32-1.39 | 0.28 |
| Race and ethnicity |  |  |  |
| White non-Hispanic | (Reference) |  |  |
| Black or Hispanic | 1.23 | 0.57-2.66 | 0.60 |
| Other | 1.08 | 0.32-3.64 | 0.91 |
| Primary insurance |  |  |  |
| Non-Medicare | (Reference) |  |  |
| Medicare | 0.42 | 0.17-1.02 | 0.06 |
| Distance to treatment facility |  |  |  |
| < 25 miles | (Reference) |  |  |
| 25-50 miles | 0.51 | 0.19-1.43 | 0.20 |
| 50-75 miles | 0.28 | 0.03-2.68 | 0.27 |
| 75-150 miles | 0.87 | 0.30-2.53 | 0.80 |
| 150-500 miles | 0.49 | 0.18-1.29 | 0.15 |
| 500-5000 miles | 0.72 | 0.25-2.07 | 0.55 |
| International | 1.09 | 0.20-5.90 | 0.92 |
| Primary cancer site |  |  |  |
| Lung and Thorax | (Reference) |  |  |
| Breast | 0.52 | 0.12-2.16 | 0.37 |
| Central nervous system | 4.26 | 0.61-29.58 | 0.14 |
| Connective and soft tissue | 2.86 | 0.46-17.94 | 0.26 |
| Gastrointestinal* | 1.38 | 0.42-4.51 | 0.60 |
| Genitourinary | 1.03 | 0.26-4.03 | 0.97 |
| Gynecologic | 0.81 | 0.08-8.65 | 0.86 |
| Head and Neck | 2.60 | 0.80-8.44 | 0.11 |
| Hematologic | 1.16 | 0.33-4.17 | 0.82 |
| Skin | 0.75 | 0.08-7.27 | 0.81 |
| Other** | 1.64 | 0.34-7.86 | 0.54 |
| Treatment for disease recurrence |  |  |  |
| No | (Reference) |  |  |
| Yes | 0.82 | 0.30-2.20 | 0.69 |
| Treatment goal |  |  |  |
| Non-palliative | (Reference) |  |  |
| Palliative | 2.77 | 1.37-5.63 | **0.005** |
| Number of radiotherapy fractions |  |  |  |
| 1-5 | (Reference) |  |  |
| 6+ | 0.88 | 0.43-1.81 | 0.73 |

Abbreviations: OR, odds ratio; CI, confidence interval

* *Includes esophagus*

** *Includes adrenal gland, bone, neuroendocrine, non-cancer, retroperitoneum and peritoneum and unspecified*

†*Includes ethnicity or race categories with counts less than 10*
